# The Registrar-General's Figures

**Published:** 1921-01-08

**Authors:** 


### THE PUBLIC WELL-BEING.

### The Registrar-General's Figures.

### SAVING THE CHILD LIFE OF THE COUNTRY.

The 1919 report of the Registrar-General for England and Wales is an interesting and on the whole encouraging document. The paragraph on which public comment has been chiefly directed seems to be the following conclusion among many others drawn by the statisticians:—

While the time is hardly ripe yet to attempt an estimate of the total loss of births attributable to the war, possibly the number may be somewhere over half-a-million, or very similar to that of deaths on active service.

It would be unwise to attach too precise a significance to this estimate, which, after all, is one made in the broadest terms and on the broadest data; but it is certainly an interesting conclusion which puts the figure considerably higher than was generally supposed. It also suggests that those who are calling out for a general system of birth control with the object of relieving the population or definitely checking its growth may be inclined to modify their enthusiasm, at any rate for the time being. According to an expert view, and in the light of past experience as far as it can be brought home, in the absence of reliable statistics, it seems to be usually the case after a considerable war that the populations of the belligerent countries do actually fall away perceptibly over a long period of years, though the decline does not make itself apparent immediately. We believe that after the Franco-German War of 1870, a trivial affair compared to the Great War, the effect on the German population was marked for more than twenty years, and that it was not until well into the nineties that the normal rate of increase was again approached.

### The Effects of War.

It is worth noting from the report that a further effect of the war is emphasised by the enormous number of young women who were created widows, and the proportion who have re-married. Over ten times as many widows under the age of twenty-five re-married as in 1911. For those from twenty-five to thirty years of age the increase was six times over the 1911 figures. The tendency is notably manifested among bachelors whose marriages at ages over forty-five were, both in 1918 and 1919, more than twice as numerous as in 1911. The general tendency towards later marriages is more pronounced than ever, and that in its turn must have a very considerable effect upon the birth-rate in the year immediately to come.

The one outstanding feature of the report which must give satisfaction and new stimulus to those who are actively concerned in improving measures of public health, and perfecting the hygiene of living in our densely crowded areas, is the general tendency for mortality at most ages to decline, though cancer is singled out as the exception among diseases over which greater control seems to be secured. It is really remarkable that in a single year there should be recordel by far the lowest death-rate ever attained from such important causes of child mortality as measles and whooping-cough, and a death-rate from diarrhœa which has only once before been bettered. The excess of London infant mortality over the average for the country, which was noted as an exceptional feature of the returns of 1917 and 1918, has disappeared, owing mainly to a remarkable fall in the London mortality at 6-12 months.

### Mortality in London.

London, however, shared in the general increase of mortality applying to the first four weeks of life, and its normal advantage at this period has been somewhat reduced. As in each of the five preceding years, it compared worse with the remainder of the country at 3-6 months than at any other part of the first year. The sentence which should be set out in deep black letters is that which points out that the excess of child mortality in the north over the south was well marked from the very first day of life. What does that mean? It means that the crucial problem of preserving the child-life of the country resides in the great industrial, crowded, dismal, dark, forbidding districts of the North of England. Here a gigantic work remains to be done.

